# Thromboelastography to Support Clinical Decision Making in Patients with Peripheral Artery Disease

**DOI:** 10.3390/diagnostics15243113

**Published:** 2025-12-08

**Authors:** Anahita Dua, Isabella Cieri, Adriana Rodriguez, Shiv Patel, Dawn Barberi, Joao D. Dias, Jan Hartmann

**Affiliations:** 1 Division of Vascular Surgery, Massachusetts General Hospital, Harvard Medical School, Boston, MA 02114, USA; 2 Massachusetts General Hospital, Boston, MA 02114, USA; 3 Global Medical Office (GMO), Haemonetics Corporation, Boston, MA 02110, USA

**Keywords:** antiplatelet therapy, antithrombotic therapy, hypercoagulability, major adverse cardiovascular events (MACE), peripheral artery disease (PAD), personalized medicine, platelet function, PlateletMapping^®^ assay, revascularization, thromboelastography (TEG^®^)

## Abstract

Peripheral artery disease (PAD) leads to reduced blood flow, primarily affecting the vessels of lower extremities. Symptoms include pain, cramping and reduced functional capacity, and patients are also at increased risk of cardiovascular complications and mortality. Postoperative medical management in PAD patients includes the use of antiplatelet and antithrombotic medications, which help to prevent postoperative graft and stent thrombosis and associated adverse effects. Despite extensive research, there is little consensus on the best strategy or medication regimen for patients with PAD or on monitoring strategies for the antithrombotic therapies. Thromboelastography, with the adjunct of platelet function assessment, is well established for providing real-time assessment of coagulation and platelet function in patients undergoing cardiovascular surgery or cardiovascular procedures. TEG^®^ PlateletMapping^®^ assays can assess hypercoagulable changes in pre- and post-intervention in cardiovascular patients, including in patients with PAD and help physicians guide antithrombotic treatments after revascularization. The use of thromboelastography with platelet function analysis provides an opportunity to tailor antithrombotic therapy and personalize care in patients with PAD, which could be integral to improving limb salvage and preventing adverse events in these patients.

## 1. Introduction

Peripheral artery disease (PAD) is a progressive disorder characterized by stenosis and/or occlusion of large and medium-sized arteries primarily affecting the vessels of lower extremities [1,2,3]. It leads to reduced blood flow that causes symptoms such as pain, cramping and reduced functional capacity and is also associated with an increased risk of cardiovascular complications and mortality [1,2,4]. Globally, more than 230 million people are estimated to be affected by PAD [5,6,7]. Its prevalence rises with age, affecting a substantial proportion of the elderly population, with over 20% of affected people older than 80 years [1]. Other well-recognized risk factors for PAD include smoking and diabetes. Diabetic patients have a 2–4× higher risk of developing PAD and have a higher risk of developing the most severe forms of PAD [1,8]. The overall prevalence of PAD is either similar or higher in women compared with men and increases with age [6]. Notably women with PAD often suffer worse outcomes than men even though they have fewer risk factors typically associated with negative outcomes [5,6]. Compared with other cardiovascular disease conditions, PAD is often underdiagnosed and therefore undertreated. The PAD population exhibits significant variability in presentation, spanning from asymptomatic to lifestyle-limiting claudication to the most severely affected patients with critical limb ischemia requiring peripheral bypass surgery to save the limb and preserve its function. Crucially, between 5 and 17% of patients experience early bypass graft and/or stent thrombosis, which is often the leading cause of amputation, major adverse cardiovascular events (MACEs) and mortality [9]. Thromboelastography (TEG^®^ 6s Hemostasis Analyzer, Haemonetics Corp., Boston, MA, USA) and thromboelastometry (ROTEM^®^ sigma coagulation testing system, Medford, MA, USA) are Viscoelastic Hemostatic Assays (VHAs). They are widely used for viscoelastic testing of whole blood samples from patients undergoing trauma, cardiac, transplant and other surgeries with high bleeding risk to optimize hemostatic therapy. The TEG^®^ 6s PlateletMapping^®^ cartridge (Haemonetics Corp., Boston, MA, USA) additionally allows for qualitative assessment of platelet function and is used to assess hemorrhage or thrombosis conditions in cardiovascular surgery and cardiology procedures [10].

## 2. Antithrombotic Therapy in Patients with PAD

Thrombosis and stenosis are key factors in PAD progression and complications, with activated platelets and hypercoagulability contributing to occlusive thrombi formation [11]. Initial treatment recommendations for patients with PAD include modification of risk factors and the use of cardioprotective medications to mitigate MACE, major adverse limb events (MALEs) and cerebrovascular events [12]. Patients with PAD can be prescribed antiplatelet agents such as aspirin, reversible and irreversible purinergic G protein-coupled receptor 12 (P2Y12) inhibitors, either as mono-antiplatelet therapy (MAPT) or as dual-antiplatelet therapy (DAPT) [11]. Protease-activated receptor 1 (PAR-1) antagonists are selectively used in addition to either MAPT or DAPT. Antithrombotic therapy is crucial after revascularization for preventing MACE and MALE such as amputation and acute limb ischemia [13]. Despite this, there is a lack of consensus for medical management in postoperative PAD. And most recommendations for patients with PAD are derived from subgroup analyses of randomized trials for patients with coronary and/or cerebrovascular disease [14]. This lack of high-quality data from randomized controlled trials in patients specifically with PAD translates to wide variations in clinical practice.

Current PAD guidelines recommend long-term MAPT with aspirin or clopidogrel alone, or dual-pathway inhibition with low-dose rivaroxaban (2.5 mg twice daily) combined with low-dose aspirin [15,16,17,18]. After endovascular revascularization procedures, DAPT with aspirin and clopidogrel is generally recommended; however, there is limited consensus for the optimal duration of the therapy. This puts patients with PAD at high risk of being undertreated with antithrombotic agents [19,20].

On top of this, individual responses to antiplatelet therapy vary significantly, with 60–65% of patients with cardiovascular disease having resistance to aspirin and/or clopidogrel [21,22]. There is also limited guidance on monitoring patient response to antithrombotic therapy in this diverse population. Current standards for assessing hypercoagulability, such as prothrombin time, international normalized ratio, and activated partial thromboplastin time, measure only the individual steps of the coagulation cascade in a non-physiologic setting (platelet-free plasma) and do not evaluate the effectiveness of commonly used antithrombotic agents in PAD [23].

Overall, the current “one-size fits all” pharmacologic approach to antiplatelet and anticoagulant therapy falls short in providing evidence-based treatment for all patients with PAD. With the risks of MALEs, mortality and bleeding being differently weighted according to the disease stage, a more personalized approach to treatment may be beneficial.

## 3. Thromboelastography Has a History of Clinical Use in Assessing Patient Hemostasis

Thromboelastography is a rapid and simple way to evaluate a patient’s coagulation status and to assess for abnormalities in platelet function and fibrinolysis in real time. It measures whole blood coagulation dynamics including clot formation, strengthening, and breakdown. Thromboelastography has been used to guide patient blood management for several decades [24], and its use is well established in patients with acute blood loss and coagulopathy in different clinical settings, including trauma [25], peri-operative bleeding [26], liver transplant [26], elective surgery [27] and cardiac surgery [28,29]. In surgical cardiac patients, use of a thromboelastography-based intraoperative transfusion algorithm has been shown to reduce the use of allogeneic blood products including fresh frozen plasma, platelets, cryoprecipitate, and red blood cells, as well as autologous transfusions from the cell saver, compared with historical controls [30].

## 4. Assessing Platelet Function with Thromboelastography Can Provide an Analysis of Platelet Contribution to Hemostasis

**Figure 1:**
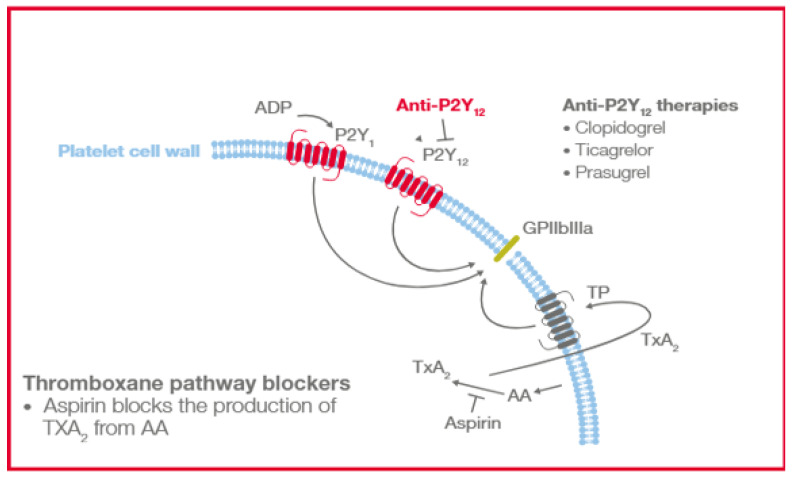
Platelet AA and ADP-activated receptors blocked by antiplatelet therapies. AA, arachidonic acid; ADP, adenosine diphosphate; GPIIbIIIa, glycoprotein I*i*b/IIIa; P2Y_12_, purinergic G protein-coupled receptor; TP, human TXA_2_ receptor; and TXA_2_, Thromboxane A_2_.

The TEG^®^ analyzer using PlateletMapping^®^ assay (Haemonetics Corp., Boston, MA, USA) can assess differential platelet response in the presence of specific platelet receptor activators and, therefore, provide an analysis of the platelet contribution to hemostasis at multiple platelet-activated receptors [31,32,33,34]. The assay evaluates platelet function through direct activation of the thromboxane pathway with arachidonic acid (AA) or P2Y12 receptor with adenosine diphosphate (ADP), allowing for the semiquantitative analysis of platelet aggregation and inhibition (Figure 1). This can be used by clinicians to help diagnose platelet function disorders; assess patients’ bleeding and thrombotic risks due to platelet function inhibition, e.g., inhibition caused by antiplatelet drugs such as aspirin, clopidogrel and others; and evaluate and monitor effectiveness of antiplatelet therapies. TEG^®^ PlateletMapping^®^ can be performed either on the TEG^®^ 5000 device through ADP and AA assay kits or the TEG^®^ 6s device using the PlateletMapping^®^ cartridge.

With thromboelastography utilization now endorsed in several international clinical guidelines [25,28,29,35], TEG^®^ assays may serve as dynamic tools across diverse cardiovascular conditions [23]. While these assays are particularly well established in postoperative setting applications, such as identifying patients at increased risk of hemorrhage and transfusion requirements, their potential utility in prothrombotic conditions has only recently emerged. The TEG^®^ device has shown potential in helping clinicians assess platelet function prior to stenting and evaluate the efficacy of antiplatelet therapies post-stent deployment, which may assist in identifying patients at increased risk for carotid plaque formation and stability [23]. Specific TEG^®^ assay thresholds may also be used to inform risk for hypercoagulability and thromboembolic events among various patient populations [23]. Measurements of platelet–fibrin clot strength and platelet reactivity have been shown to be independent predictors of clinical prognosis and long-term ischemic events post-percutaneous coronary intervention (PCI) and could serve as useful tools to improve post-PCI risk stratification for personalized antithrombotic treatment [36,37,38].

The TEG^®^ PlateletMapping^®^ assays compare well with whole blood platelet function tests, such as Multiplate^®^ (Roche Diagnostics, Rotkreuz, Switzerland) and VerifyNow^®^ (Werfen, Barcelona, Spain) under standardized in vitro conditions [33]. In addition, TEG^®^ PlateletMapping^®^ assays have also been evaluated in clinical trials to assist physicians in identifying patients undergoing coronary stenting who are at increased risk of MACE and bleeding events [39].

## 5. Utility of TEG^®^ PlateletMapping^®^ Testing Technology in Patients with PAD

**Figure 2:**
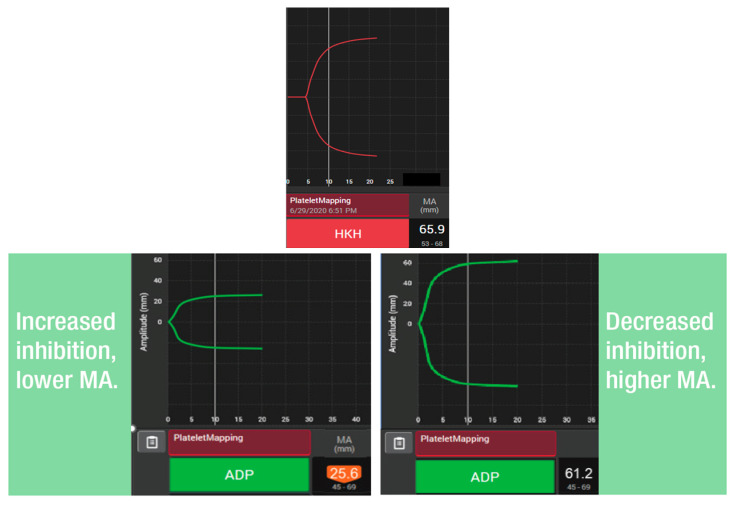
Use of TEG^®^ PlateletMapping^®^ testing: (upper panel) assessing the patient’s underlying, uninhibited clot strength (HKH-MA) and (lower panels) measuring platelet receptor function stimulated by ADP agonists (ADP MA). ADP, adenosine diphosphate; HKH, kaolin with heparinase; and MA, maximum amplitude.

**Figure 3:**
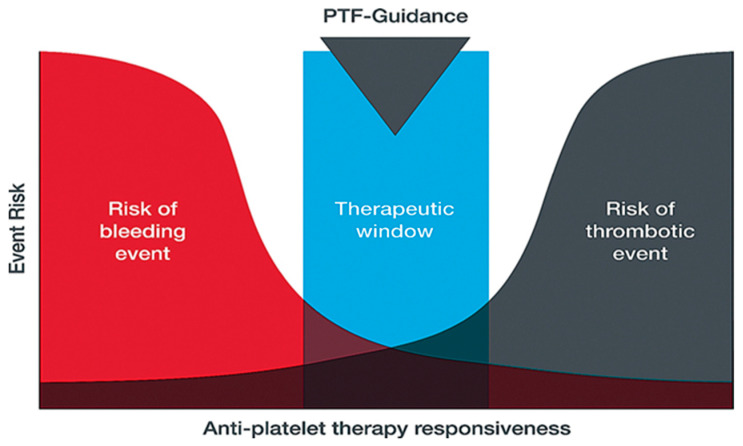
Use of platelet-function tests for guiding and personalizing balance of antiplatelet therapy based on platelet responsiveness. PTF-guidance, platelet function guidance.

The TEG^®^ PlateletMapping^®^ assay simultaneously provides insight into baseline platelet function status via the kaolin maximum amplitude (MA) (i.e., the patient’s maximal platelet function in the absence of the inhibiting drugs), alongside the current response to P2Y12 receptor inhibitors and cyclooxygenase-1 (COX-1) inhibitors via the ADP and the AA tests, respectively (i.e., demonstrating the clot strength when platelets are activated solely through those specific receptors) (Figure 2). The kaolin MA has shown the ability to identify patients at increased risk of adverse events independent of ADP and AA-induced response and may be a potential screening tool to uncover patients who would benefit from enhanced therapy or closer monitoring [38,40]. Additionally, the kaolin MA could be used to navigate treatment decisions including choice of therapy, and escalation/de-escalation decisions [38]. Thus, assessing platelet function with TEG^®^ PlateletMapping^®^ could potentially help with clinical decision making in patients with PAD by providing comprehensive coagulation metrics that aid patient-centered thromboprophylaxis [41]. For example, in the context of PCI procedures, antiplatelet therapy de-escalation strategies guided by platelet function testing have been included in the guidelines for acute coronary syndrome patients undergoing PCI as an alternative approach to 12 months of potent platelet inhibition, especially for patients deemed unsuitable for sustained potent platelet inhibition [42]. A potential utility of the TEG^®^ PlateletMapping^®^ assay, therefore, is in monitoring an individual’s response to antiplatelet therapy to identify the ideal therapeutic window between the risks of bleeding and thrombosis (Figure 3).

Promising research has supported the use of direct oral anticoagulants in patients with PAD [43,44], in part based on their theoretical dual-pathway attenuation of both thrombin generation and platelet aggregation. In patients with low responsiveness to clopidogrel, as measured by thromboelastography, intensified antiplatelet strategies with adjunctive use of cilostazol might significantly improve clinical outcomes without increasing the risk of major bleeding [45].

Overall, the TEG^®^ PlateletMapping^®^ assay could offer clinicians valuable insights to potentially guide informed decisions about medication dosages and combinations. While previous trials exploring the use of other platelet function tests to optimize outcomes among patients with acute coronary syndromes and cardiac stents have largely failed to correlate with decreased complications [46,47,48], a similar study has never been conducted in postoperative patients with PAD or with TEG^®^. TEG^®^ PlateletMapping^®^ may provide insight into the coagulation profile of patients with PAD and could be utilized to identify prothrombotic states [9,49]. In patients undergoing lower extremity revascularization, platelet reactivity is predictive of subacute postoperative graft or stent thrombosis [9]. Alternative or augmented antithrombotic regimens for high-risk patients, defined on the basis of platelet aggregation (cut off >70.8%) and platelet inhibition (cut off <29.2%), could be considered to mitigate the risk of postoperative thrombotic events [9]. Additionally, prothrombotic viscoelastic coagulation measurement with other TEG^®^ assays could help assess infection and poor wound healing following lower extremity revascularization [49]. High-risk patients identified based on cut-off points may also benefit from an enhanced antimicrobial and/or antithrombotic treatment approach [49].

Notably, TEG^®^ PlateletMapping^®^ testing has also been used to help physicians describe sex dimorphism in platelet reactivity between male and female patients with PAD undergoing revascularization [50,51]. Female patients show higher platelet reactivity—with increased platelet aggregation and diminished platelet inhibition—compared with male patients, despite similar antiplatelet management. This was associated with an increased incidence of thrombosis after revascularization [50]. Therefore, personalized antiplatelet therapy based on precise coagulation assays could mitigate sex-specific outcome disparities caused by inadequate thromboprophylaxis in women [52]. A recent study by Suarez et al. [52] showed for the first time that use of an algorithm to tailor antiplatelet medication in patients with PAD post-revascularization significantly decreased thrombotic event rates. Overall, these findings have the potential to translate to improved individualized care for men and women with, or at risk for, vascular disease.

## 6. Conclusions

Thromboelastographic analysis, with the adjunct of platelet function assessment, is well established for providing real-time assessment of coagulation and platelet function in cardiovascular patients. Over the past decade, the clinical utilization of TEG^®^ testing in cardiovascular patients has evolved greatly, and there is a growing body of evidence informing potential clinical utility of TEG^®^ technology with the PlateletMapping^®^ assay in the setting of PAD. Potential applications include the assessment of the responsiveness to platelet therapy to better inform time course analyses of hypercoagulable changes pre- and post-intervention in patients with PAD and guiding the optimal antithrombotic regimen after revascularization. Early research is exploring TEG^®^ based algorithmic antiplatelet coagulation treatment in PAD patients [52]. Thromboelastography with platelet function analysis could thus provide an opportunity for personalized and quantitative antithrombotic strategies that play a crucial role in preventing adverse events and enhancing limb salvage rates in patients with PAD.

## Data Availability

No new data were created or analyzed in this study. Data sharing is not applicable to this article.

## Abbreviations

The following abbreviations are used in this manuscript:

AA	Arachidonic acid
ADP	Adenosine diphosphate
COX-1	Cyclooxygenase-1
DAPT	Dual-antiplatelet therapy
GPIIbIIIa,	Glycoprotein IIb/IIIa
HKH	Kaolin with heparinase
MA	Maximum amplitude
MACE	Major adverse cardiovascular event
MALE	Major adverse limb event
MAPT	Mono-antiplatelet therapy
P2Y_12_	Purinergic G protein-coupled receptor
PAD	Peripheral artery disease
PAR-1	Protease-activated receptor 1
PCI	Percutaneous coronary intervention
TP	Human TXA_2_ receptor
TXA_2_	Thromboxane A_2_
